# Characterising a PDMS based 3D cell culturing microfluidic platform for screening chemotherapeutic drug cytotoxic activity

**DOI:** 10.1038/s41598-020-72952-1

**Published:** 2020-09-28

**Authors:** M. Ibrahim Khot, Mark A. Levenstein, Greg N. de Boer, Gemma Armstrong, Thomas Maisey, Hafdis S. Svavarsdottir, Helen Andrew, Sarah L. Perry, Nikil Kapur, David G. Jayne

**Affiliations:** 1 Leeds Institute of Medical Research at St. James’s, St. James’s University Hospital, University of Leeds, Beckett St., Leeds, West Yorkshire, LS9 7TF UK; 2 Department of Mechanical Science and Engineering, University of Illinois at Urbana-Champaign, Urbana, IL 61801 USA; 3 School of Mechanical Engineering, University of Leeds, Leeds, LS2 9JT UK

**Keywords:** Cancer, Cell biology, Physiology, Engineering, Nanoscience and technology

## Abstract

Three-dimensional (3D) spheroidal cell cultures are now recognised as better models of cancers as compared to traditional cell cultures. However, established 3D cell culturing protocols and techniques are time-consuming, manually laborious and often expensive due to the excessive consumption of reagents. Microfluidics allows for traditional laboratory-based biological experiments to be scaled down into miniature custom fabricated devices, where cost-effective experiments can be performed through the manipulation and flow of small volumes of fluid. In this study, we characterise a 3D cell culturing microfluidic device fabricated from a 3D printed master. HT29 cells were seeded into the device and 3D spheroids were generated and cultured through the perfusion of cell media. Spheroids were treated with 5-Fluorouracil for five days through continuous perfusion and cell viability was analysed on-chip at different time points using fluorescence microscopy and Lactate dehydrogenase (LDH) assay on the supernatant. Increasing cell death was observed in the HT29 spheroids over the five-day period. The 3D cell culturing microfluidic device described in this study, permits on-chip anti-cancer treatment and viability analysis, and forms the basis of an effective platform for the high-throughput screening of anti-cancer drugs in 3D tumour spheroids.

## Introduction

The interest in and practicality of microfluidic systems for biochemical analysis of living cells and tissues has dramatically increased over the past decade^1,2^. ‘Lab-on-a-chip’ technologies carry many advantages, including the convenience of culturing cells and performing analytical techniques on small microfabricated platforms. Consequently, these devices consume only a fraction of the amount of reagents, tissue culturing media and precious biological specimens as do conventional in vitro experiments that are carried out in large commercial plates and flasks^3^. Another advantage of microfluidic devices for biomedical research is their ability to streamline complex assay protocols^4^. For instance, fluid flow enables the culture and treatment of cells to be spatially and temporally controlled with a great degree of accuracy^5^. Specifically for anti-cancer research, microfluidic systems have the potential to quickly and easily screen drugs in real time and diagnose disease^6^.

Concurrent with the increasing practical application of microfluidics in the biomedical sciences, three-dimensional (3D) cell culturing is increasingly being adopted in anti-cancer research, especially for the purpose of drug development. In comparison to conventional two-dimensional (2D) monolayer cell cultures, 3D spheroids are recognised as better in vitro models of cancer due their inherent properties, making them more comparable to tumour in vivo^7^. Cells in 2D cultures are uniform in their rate of proliferation, access to oxygen and nutrients and response to treatment, whereas the architectural arrangement of cells in 3D spheroids, together with the physiological gradients of oxygen, nutrients, growth factors and catabolites results in the uneven growth and diverse survival rates of cells. In addition, the layered structure of cells influences the ability of anti-cancer therapeutics to penetrate through and induce cytotoxicity in the different regions of the spheroid. Cells cultured in a 3D structure also experience different mechanical and topographical forces, than those growing on a 2D planar surface^8^. The overall benefit of 3D spheroidal models is that they offer a better understanding of in vivo molecular mechanisms involved in tumour growth and drug resistance^9^.

Taken together, 3D spheroids offer a better representation of in vivo environments and microfluidic devices offer convenient and cost-effective platforms for high-throughput screening applications. A common limitation among established 3D cell culturing techniques is the manual effort required to grow and maintain 3D spheroid cultures in non-specialised conventional commercial tissue culturing equipment. An appropriately designed microfluidic platform can improve the success rate of establishing and characterising 3D spheroids by reducing the amount of effort required for cell culturing, administering cytotoxic treatments and evaluating the outcomes of treatment—conveniently within a single device^5,10–16^.

In this article, we describe the development and application of a 3D cell culturing microfluidic flow platform and the use of 3D printing to create a master from which polydimethylsiloxane (PDMS) flow chips can be conveniently cast. We demonstrate the platform by culturing 3D spheroids, treating with the anti-cancer drug, 5-Fluorouracil (5-FU) and evaluating cytotoxicity through flow. We show that the 3D cell culturing microfluidic platform we have developed can accommodate the generation of 3D spheroids from cell aggregates ‘on-chip’, administer cytotoxic treatment under limited- and continuous perfusion conditions and perform quantitative and qualitative cell viability analysis.

## Results

**Figure 1:**
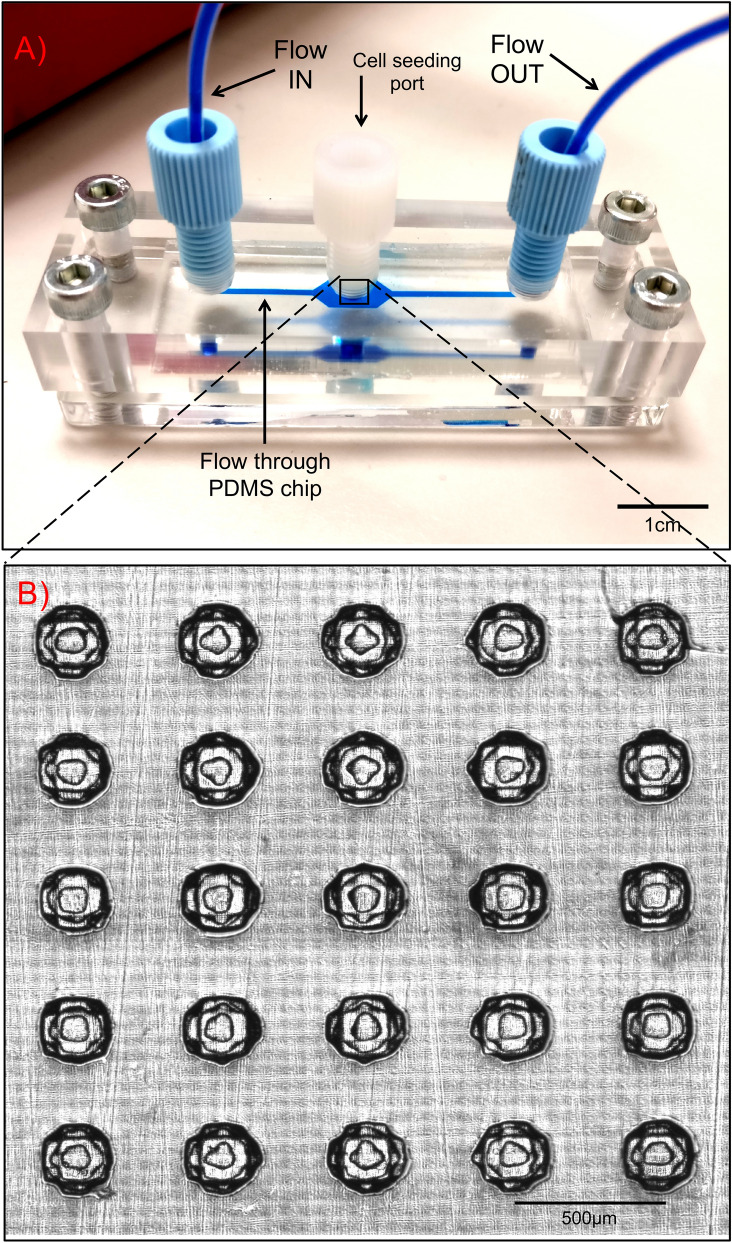
Designing the microfluidic device. The PDMS 3D cell culturing microfluidic device consisted of a central 3D cell culturing PDMS chip and is sandwiched in between two layers of PMMA. The inverse design of the PDMS chips was printed as a reusable master mould in a photocurable acrylonitrile butadiene styrene thermoplastic polymer. The PMMA layers were machined from cast sheet, with inlet and outlet ports positioned to match the ends of the PDMS chip and machined to take standard UNF 1/4-28 flangeless fittings for the two ports for flowing fluids through the device. The device was held together using four bolts with one in each corner. (**A**) Photographic image of the microfluidic device with a blue dye flowing through device and over the 3D spheroid culturing wells. Scalebar = 1 cm. (**B**) Transillumination microscopic image of the 3D cell culturing wells in the center of PDMS chip which could accommodate the simultaneous culturing of twenty-five 3D spheroids in a 5 × 5 array. Scalebar = 500 μm.

**Figure 2:**
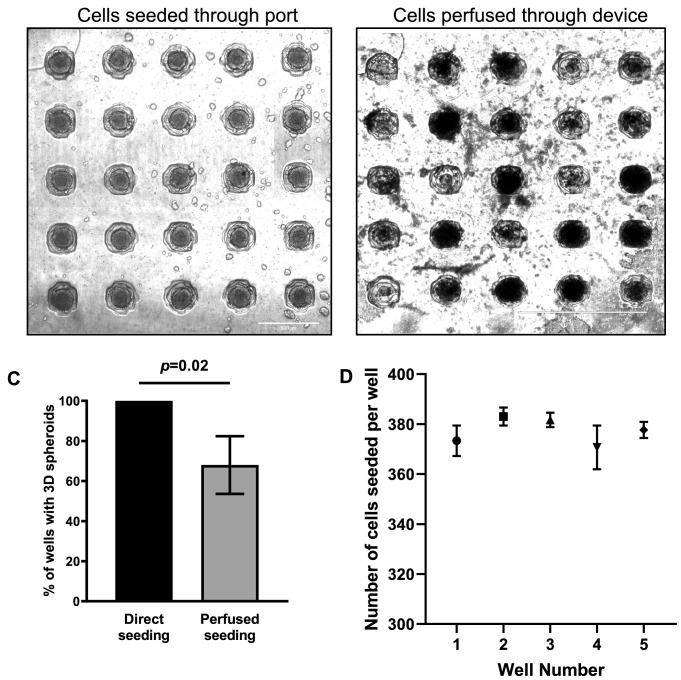
The efficiency in seeding cells and generating 3D spheroids between the addition of cells through the seeding port and perfusing cells through the microfluidic device. Transillumination images of HT29 cells in suspension that were (**A**) added directly through the top cell seeding port and (**B**) seeded through fluid flow into the microfluidic device. For both conditions, the microfluidic devices were kept under static conditions for 48 h to allow 3D HT29 spheroids to form. This demonstrates the importance of seeding cells directly to the location of the wells rather than through flow. Images are representative of at least 3 independent experiments. (**C**) Seeding cells directly into the wells is more effective at successfully culturing 3D spheroids in all the wells of the microfluidic device as compared to seeding cells through flow (3 replicates per experiment). (**D**) 20 μL of HT29 cells (2 × 10^7^cells/mL) were seeded through the top mounted cell seeding port using a pipette. 5 wells from each 5 × 5 array of wells were randomly selected and the number of cells seeded into the wells were manually counted (5 wells/replicates per experiment). Data shown represent means with standard deviation of 3 independent experiments for both spheroid counting and cell counting experiments.

**Figure 3:**
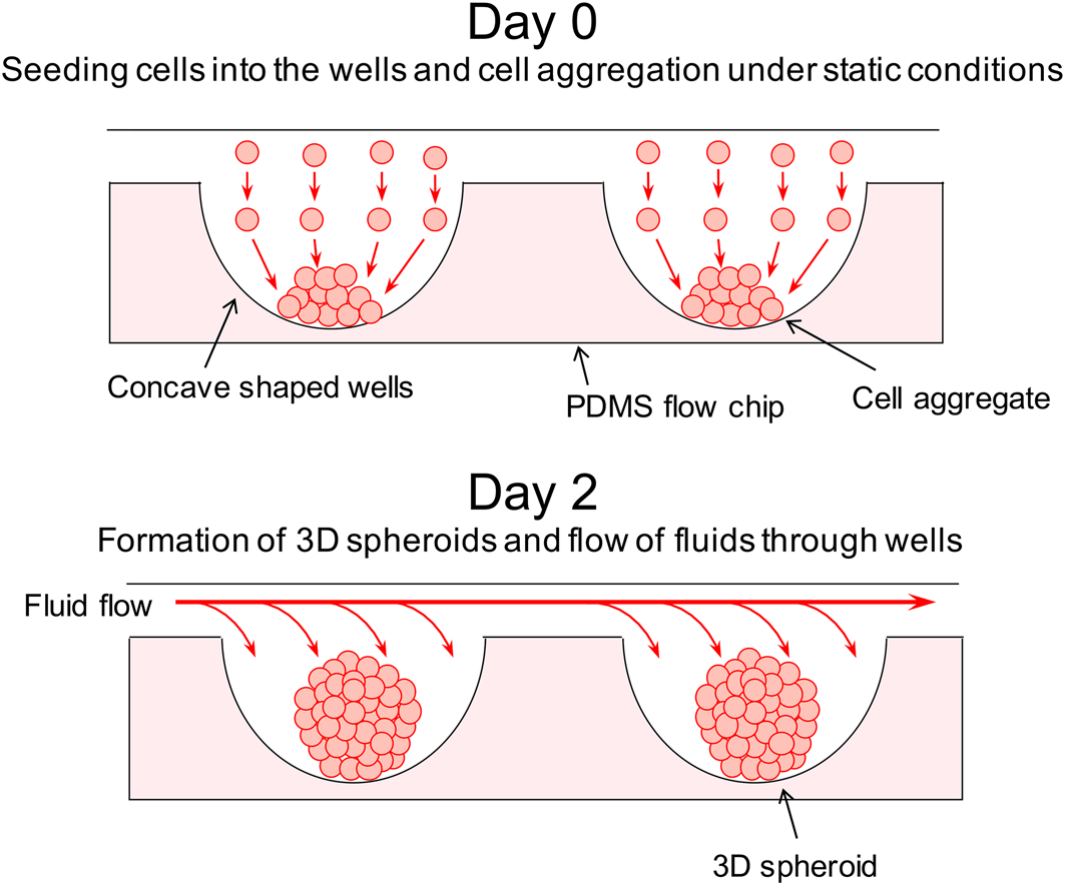
3D cell culturing wells in the PDMS flow chips. The concave shaped wells in the PDMS flow chips were designed to assist in the aggregation of cells following the deposit of HT29 cells in suspension. On Day 0 cells would be added to the wells forming cell aggregates which would then be cultured under static conditions for another 2 days to form 3D spheroids. Fluid flow would then be applied through the wells following the formation of 3D spheroids.

The microfluidic device described in this study utilises the non-cell adherent properties of the PDMS flow chip located between two layers of Poly(methyl methacrylate) (PMMA), to generate 3D spheroids (Fig. 1A)^17^. Placing a cell seeding port directly above the cell culturing wells was vital to improving the efficiency of introducing cells to the wells. The port was added after it was identified that perfusing a HT29 cell suspension through the device to seed the wells was not effective (Fig. 2A,B). Seeding cells through direct deposition via the cell seeding port, results in 3D spheroids forming in 100% of the wells, as compared to 68% using flow-driven cell seeding (*p* = 0.02) (Fig. 2C). An equivalent number of HT29 cells, (mean = 377 cells, min. = 361 cells, max. = 386 cells), were deposited into each well to generate 3D spheroids (Fig. 2D). The central region of the PDMS flow chip was designed with a 5 × 5 array of concave-shaped spheroid culturing wells (Fig. 1B). The concave-shaped wells were designed to aid in the cell seeding process, by assisting cells in initially forming cell aggregates, which is vital for successfully generating 3D spheroids (Fig. 3).

**Figure 4:**
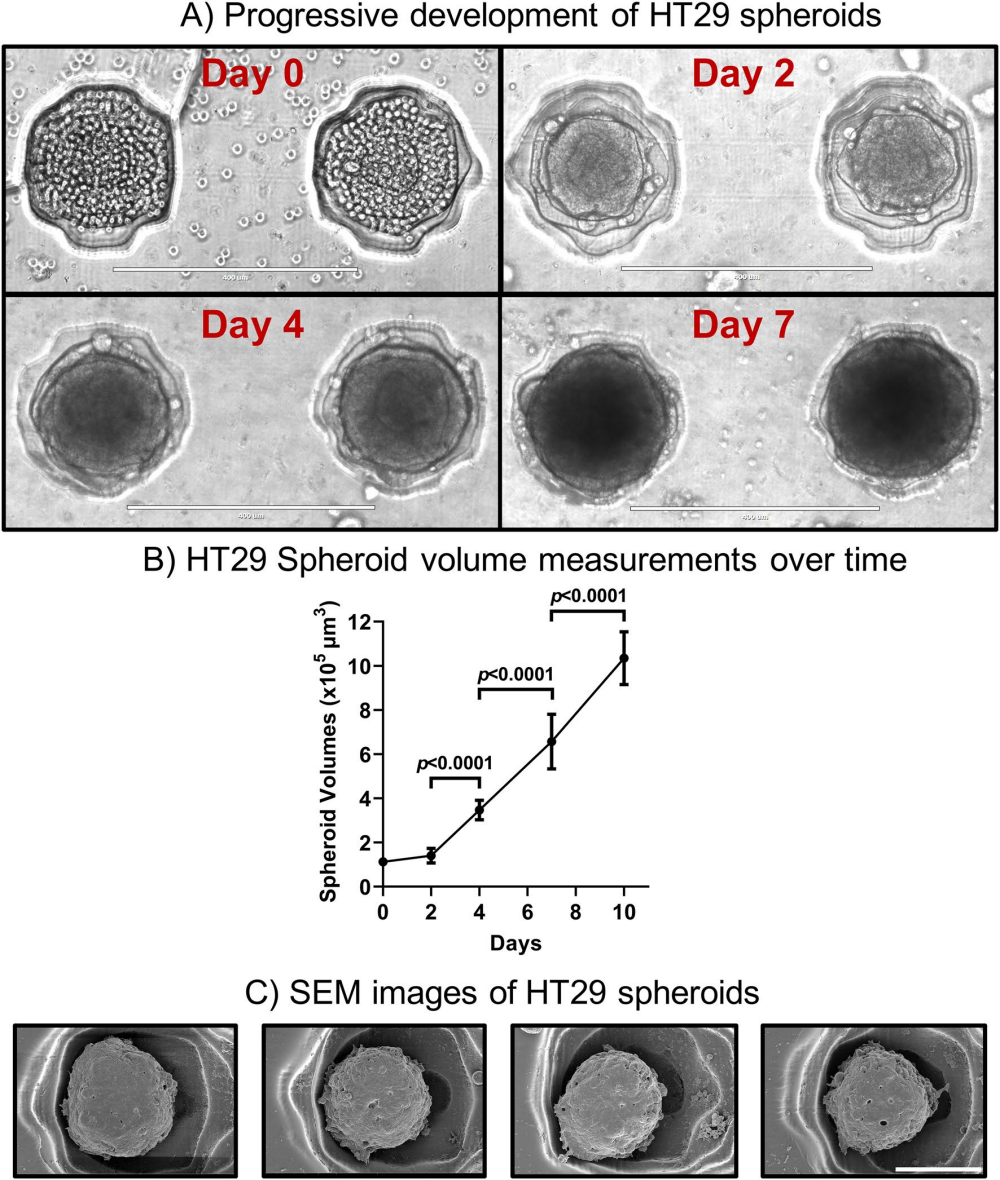
Culturing spheroids in the microfluidic flow chip. (**A**) HT29 cells were prepared in cell suspension and seeded into the spheroid culturing wells of the PDMS chips and cultured under static conditions in the PDMS chips. Transillumination microscopic images of 3D spheroids were taken showing the progressive development of 3D HT29 spheroids at Days 0, 2, 4 and 7. Scalebar = 400 μm. (**B**) HT29 cells in suspension were seeded onto the PDMS flow chips and cultured under static conditions for 10 days. Spheroid volume measurements were taken at Days 0, 2, 4, 7 and 10 to show the progressive growth of spheroids over time in the microfluidic devices**.** Data represents means with standard deviation of 3 independent experiments (3 spheroids per experiment). (**C**) 3D HT29 spheroids were cultured under static conditions in the PDMS flow chips for 10 days. Spheroids were subjected to ethanol gradient dehydration and coated with 4 nm of iridium and then imaged via scanning electron microscopy (SEM). Scalebar = 100 μm. Images are representative of at least 3 independent experiments.

As depicted in Fig. 4A, after seeding the HT29 cells into the wells of the PDMS flow chip, cell aggregates formed within a day (Day 0) and subsequent culturing under static conditions, resulted in 3D spheroids forming by Day 2. The HT29 spheroids then continued to mature in density, roundness in spheroidal structures and grew in overall size. A 148% (*p* < 0.0001), 89% (*p* < 0.0001) and 58% (*p* < 0.0001) increase in spheroid volumes were found between Day 2 versus Day 4, Day 4 versus Day 7 and Day 7 versus Day 10 respectively (Fig. 4B). Following 10 days of culture, scanning electron microscopy confirmed the spheroidal structures of the HT29 spheroids (Fig. 4C).

**Figure 5:**
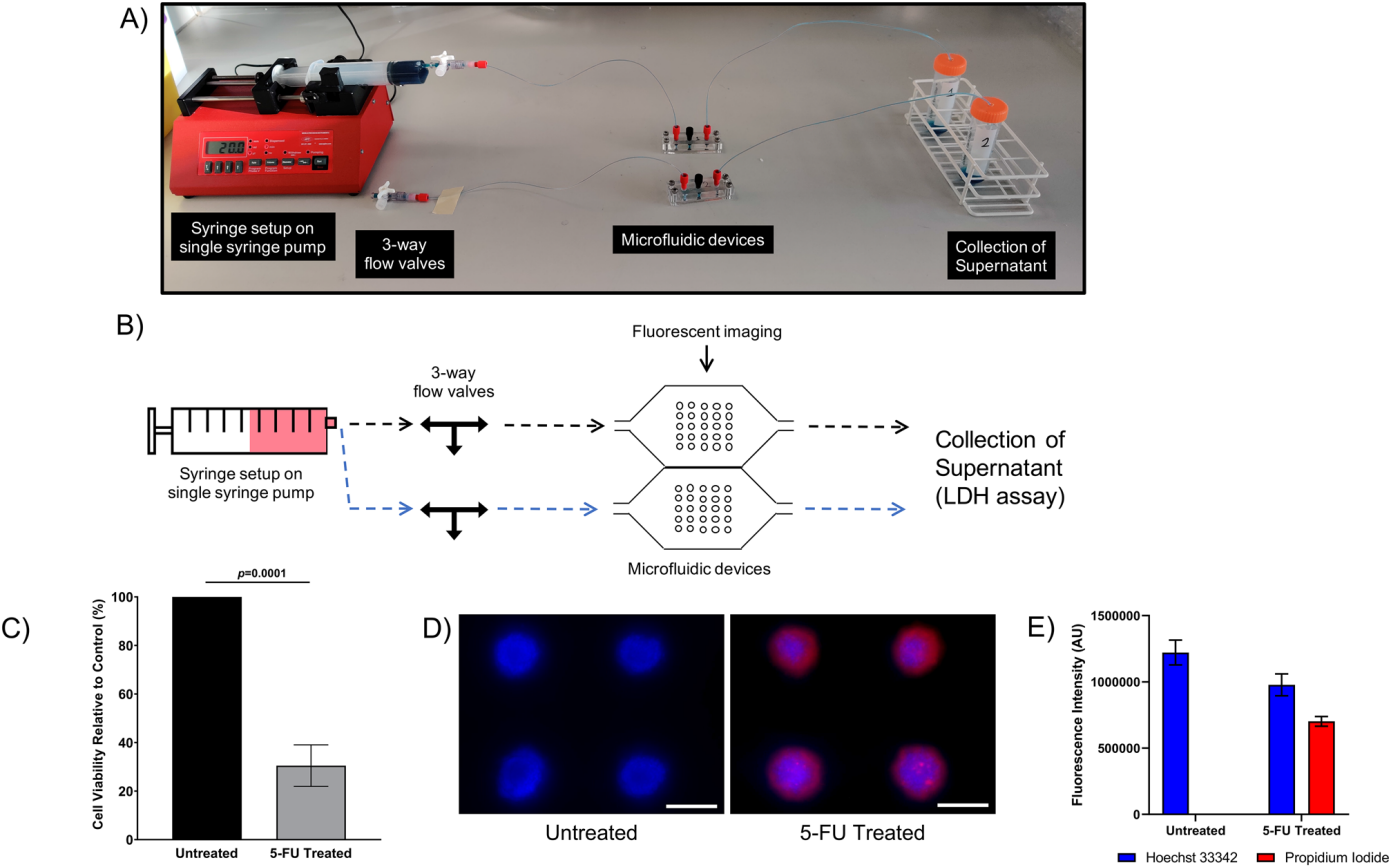
Culturing, treating and evaluating anti-cancer activity in spheroids through limited-perfusion microfluidic flow. (**A**) Photographic image and (**B**) schematic diagram of the limited-perfusion microfluidic setup using a single syringe pump. A fluid filled syringe is placed into the pump which is connected to an adjustable 3-way flow valve. The valves are connected to microfluidic devices and through to universal tubes for the collection of supernatants. During fluid flow the 3-way valve is open. Once complete perfusion through microfluidic devices has occurred the pump is stopped, and the 3-way valve is closed to create a vacuum and statically hold fluids in the microfluidic devices. HT29 cells in suspension were seeded and cultured under static conditions in the microfluidic devices flow chip for 2 days to form 3D spheroids. 3D spheroids were then treated with 5-Fluorouracil (5-FU, 500 µM) under fluid flow for 25 min at 20 µL/min and then incubated for 24 h under static conditions. (**C**) The supernatant was then collected through fluid flow and plated into 96-well plates and the Lactate dehydrogenase (LDH) assay was performed. Data are shown relative to control treated cultures and represent means with standard deviation of 3 independent experiments (3 replicates per experiment). (**D**) Spheroids were also stained with Hoechst 33342 (Blue) and propidium iodide (Red) fluorescent dyes through flow and fluorescently imaged. Scalebar = 200 μm. Images are representative of at least 3 independent experiments. (**E**) Hoechst 33342 and propidium iodide fluorescent intensity from 3D HT29 spheroids was digitally quantified using ImageJ. Data represents means with standard deviation of 3 independent experiments (3 spheroids per experiment).

Using the microfluidic platform, further experiments were carried out to highlight the (1) administration of an anti-cancer drug to 3D HT29 spheroids and (2) evaluate cytotoxicity induced by the anti-cancer drug through the microfluidic device. For limited-perfusion experiments, a single syringe pump was connected to the microfluidic devices and was used to drive flow (Fig. 5A,B). Briefly, 3D HT29 spheroids were generated on chip and subjected to 5-FU treatment (500 µM) through perfusion. After 24 h under non-flow static conditions, cell viability analysis showed that 5-FU treated HT29 spheroids were 70% less viable, as compared to untreated spheroids (*p* = 0.0001) (Fig. 5C). Fluorescent imaging highlighting cell death confirmed cytotoxicity in 5-FU treated spheroids, as indicated by the uptake of propidium iodide and lack of dye uptake in untreated HT29 spheroids (Fig. 5D). Quantifying propidium iodide fluorescence also confirmed cytotoxicity in 5-FU treated spheroids with no toxicity detected in untreated spheroids (*p* < 0.0001) (Fig. 5E). In addition, a 20% reduction in Hoechst 33342 fluorescence in treated spheroids, as compared to untreated spheroids (*p* = 0.028) indicated the loss and shedding of cells following 5-FU treatment (Fig. 5E).

**Figure 6:**
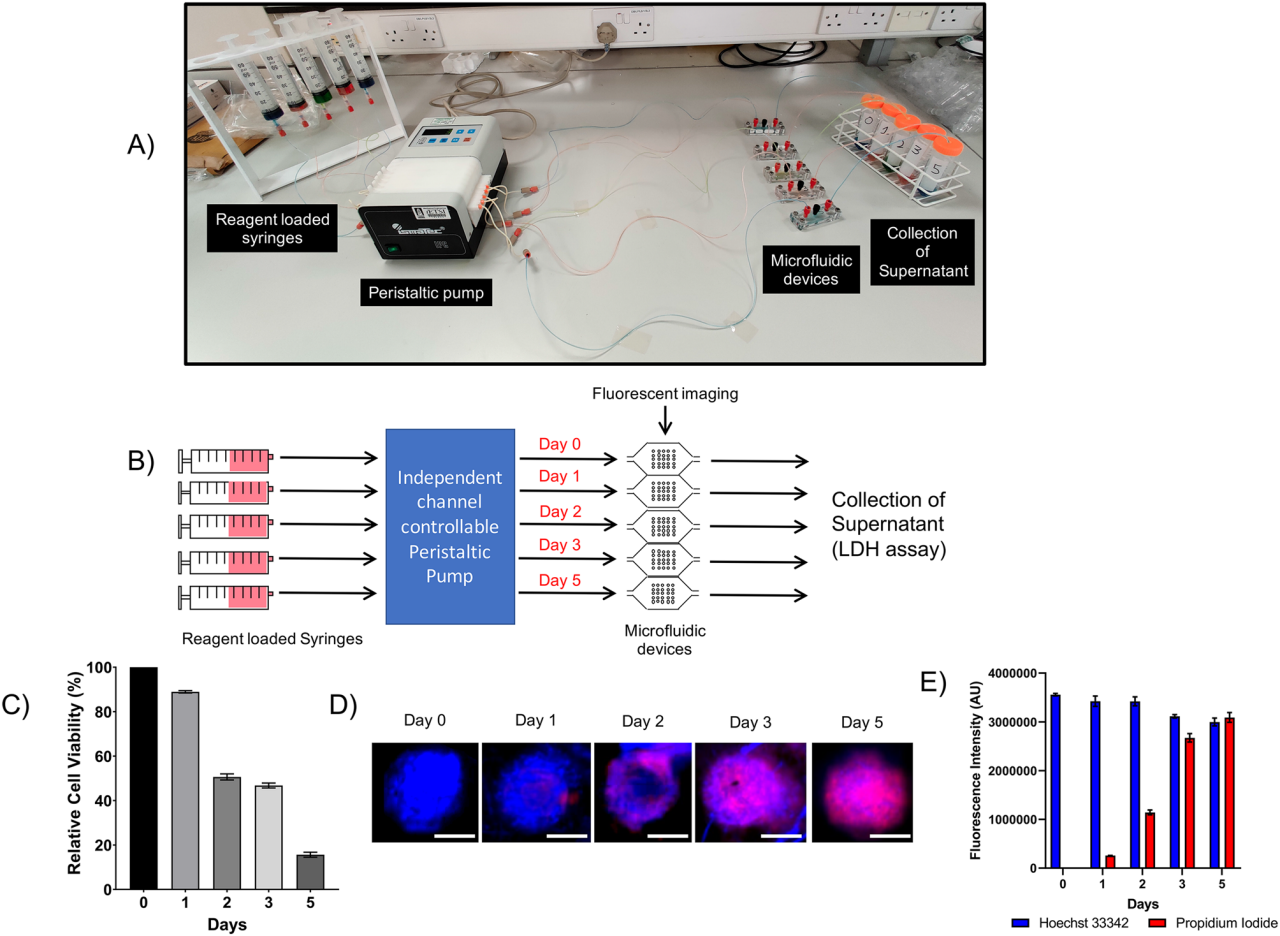
Time course monitoring of chemosensitivity in spheroids through continuous microfluidic flow. (**A**) Photographic image and (**B**) schematic diagram of the continuous perfusion microfluidic setup using peristaltic pump. Fluid filled syringes were connected to the peristaltic pump that was connected to the microfluidic devices and through to universal tubes for the collection of supernatants. HT29 cells in suspension were seeded and cultured under static conditions in the microfluidic devices flow chip for 2 days to form 3D spheroids. Spheroids were then treated with 5-FU (200 μM) through continuous perfusion at 20 µL/min for 0, 1, 2, 3 and 5 days. The independent channel controllable pump allowed flow through devices to be independently controlled permitting flow to stop through one device without influencing flow through other devices. (**C**) The supernatant was collected at the different time-points through fluid flow and plated into 96-well plates and the Lactate dehydrogenase (LDH) assay was performed. Data are shown relative to cell media control and represent means with standard deviation of 3 independent experiments (3 replicates per experiment). (**D**) Spheroids were also stained with Hoechst 33342 (Blue) and propidium iodide (Red) fluorescent dyes through flow and fluorescently imaged. Scalebar = 100 μm. Images are representative of at least 3 independent experiments. (**E**) Hoechst 33342 and propidium iodide fluorescent intensity from 3D HT29 spheroids was digitally quantified using ImageJ. Data represents means with standard deviation of 3 independent experiments (3 spheroids per experiment).

Microfluidic device-cultured HT29 spheroids were also subjected to 5-FU treatment (200 µM) through continuous perfusion. The microfluidic devices were connected in parallel to reagent-loaded syringes through a peristaltic pump (Fig. 6A,B). Cell viability was monitored over 5 days and a time dependant reduction in cell viability was observed (*p* < 0.0001) (Day 0 = 100% cell viability and Day 5 = 15% cell viability, *p* < 0.0001) (Fig. 6C). Fluorescent imaging confirmed results of the LDH assay, as indicated by the increasing uptake of propidium iodide over time (Fig. 6D). In addition, quantifying propidium iodide fluorescence also showed significant cytotoxicity over the 5 days (Day 0 vs. Day 5: *p* < 0.0001) (Fig. 6E). A 16% reduction in Hoechst 33342 fluorescence was also observed at Day 5, as compared to Day 0, indication the loss and shedding of cells (*p* = 0.0003) (Fig. 6E).

## Discussion

Three-dimensional in vitro spheroidal models of cancer are recognised as more accurate representations of the tumour microenvironment as compared to 2D monolayers, and responses to treatment in 3D cultured cells are more reflective of responses observed in vivo^8^. Combined microfluidics and 3D spheroid culturing has allowed conventional laborious 3D cell culturing protocols to be scaled down onto custom micro-fabricated devices. This allows the generation, culturing, treatment and analysis of 3D spheroids to be performed conveniently on-chip through fluid perfusion^18–23^, with the additional practical advantages of reduced experimental and consumables costs and reduced manual labour^23^.

**Figure 7:**
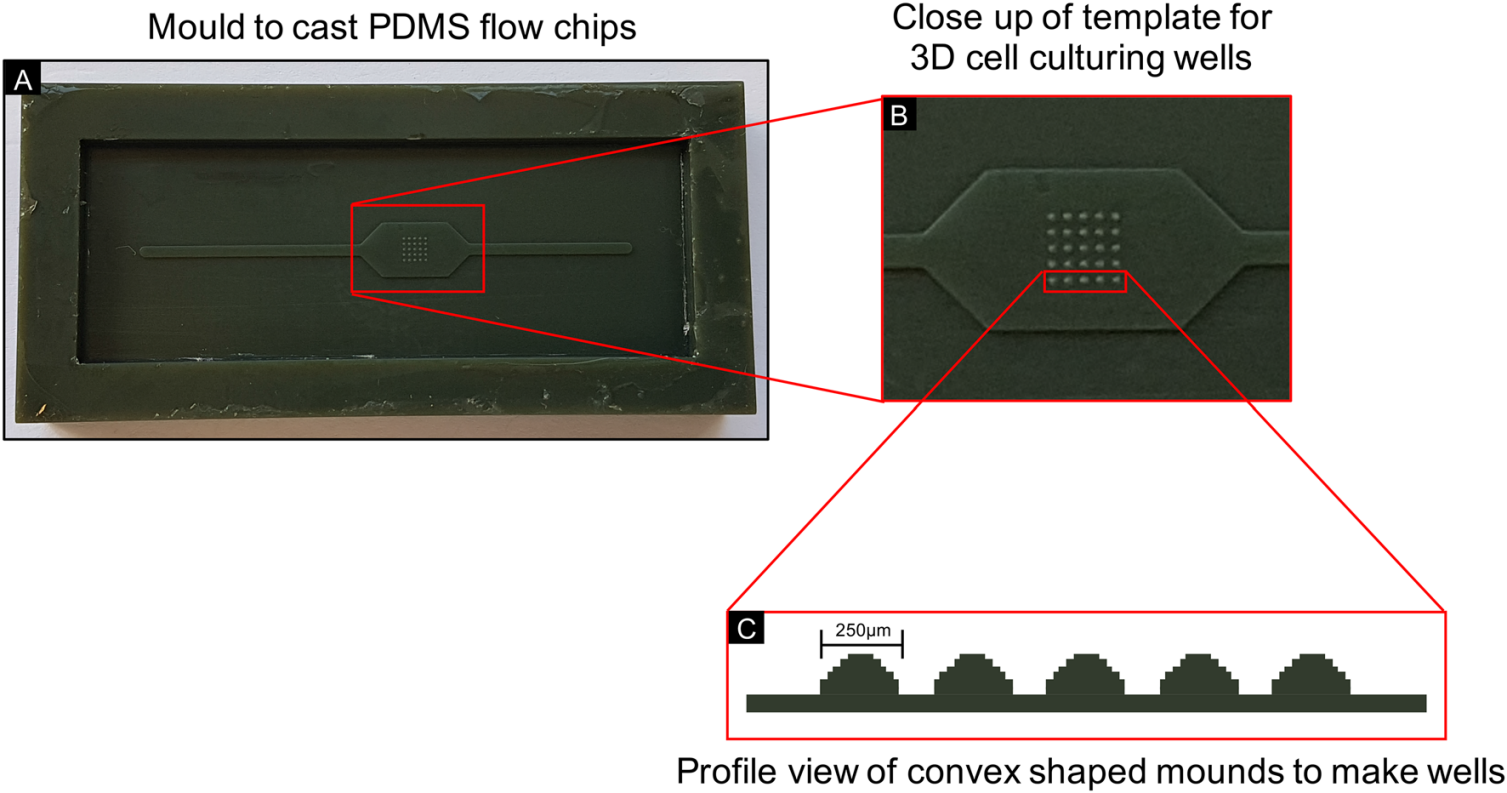
The mould used to cast the PDMS flow chips. (**A**) PDMS chips were created by casting using a mould that was 3D printed using photocurable acrylonitrile butadiene styrene with the inverse designs of the PDMS chips and (**B**) 3D cell culturing wells (250 µm in diameter) located in the middle of the mould in a 5 × 5 array on a pitch of 500 µm x 500 µm. (**C**) The layered printing to make the mould resulted in ‘step-like’ convex shaped mounds for the 3D cell culturing wells.

The 3D cell culturing microfluidic device described in this paper, builds upon previous work and is based upon the ‘non-cell adherent’ technique for generating 3D spheroids^10,24–27^. Cells are seeded on a non-adherent substrate, preventing them from adhering to the surface and instead forming cell aggregates^26^. The PDMS flow chip used for our microfluidic device is a non-cell adherent substrate, biocompatible, facilitates the exchange of gases and has been used extensively for fabricating 3D cell culturing microfluidic platforms^5,11,12,14–16,18–21,27–41^. SU-8 based microfluidic platforms are also commonly used for 3D cell culturing systems, however the chemical treatment of SU-8 to produce hydrophobic surfaces requires dangerous and toxic chemicals to be used^42^. We employed 3D printing and PDMS casting, which does not require surface treatment or bonding to other materials, making the fabrication of the flow chips inherently simple. The flow chips were cast from a mould designed with concave-shaped spheroid-culturing wells to facilitate the aggregation of cells and assist in the generation of 3D spheroids, as has been described previously for 3D cell culturing platforms^43,44^. Due to the nature of the photocuring method for forming the 3D prints, the master mould is built in layers forming ‘steps-like’ structures as an approximation to the convex well to make the 3D cell culturing wells (Fig. 7). These structures were nevertheless still successful in generating spheroids in the polygonal concave-shaped wells. Our method for making 3D spheroid culturing chips is effective, yet simple enough to allow biologists and non-microfluidics specialists to produce and operate these chips in their laboratories without the need of a cleanroom or delicate microfabrication/assembly steps. The PDMS-based spheroid culturing chip was designed to accommodate the culture of twenty-five spheroids (Fig. 1B). As a prototype design, we limited culturing to twenty-five spheroids, yet retain reliability and simplicity in generating 3D spheroids, as compared to conventional 96-well plates. However, the advantage of our design is such that the 5 × 5 array of spheroid-culturing wells, could easily be expanded to accommodate the simultaneous culturing of larger numbers of 3D spheroids.

**Figure 8:**
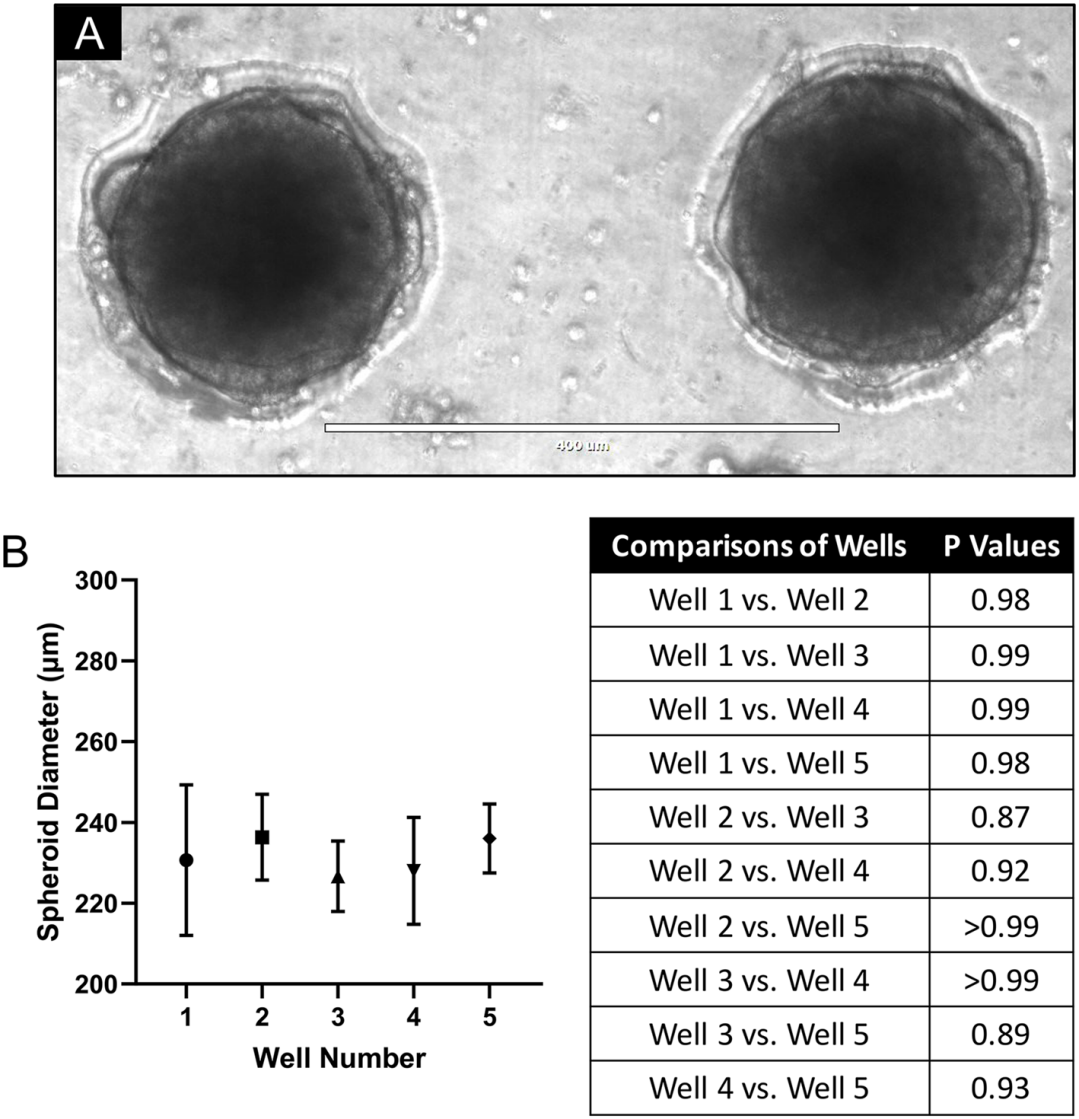
Uniform sized 3D HT29 spheroids were formed in the microfluidic device. (**A**) Transillumination microscopic image and (**B**) spheroid diameter measurements of HT29 cells in suspension that were seeded onto the PDMS flow chips and cultured under static conditions for 7 days forming uniform sized spheroids. Data shown represent means with standard deviation of 3 independent experiments (3 replicates per experiment). Images are representative of at least 3 independent experiments.

A limitation of previously reported 3D cell culturing microfluidic platforms is that they have relied on external 3D cell culturing methods, such as the use of ultra-low attachment plates or the hanging drop method, to generate spheroids off-chip before being introduced into the microfluidic platforms^5,13,35,36,40^. The microfluidic platform described in this study eliminates the need for generating 3D spheroids beforehand and allows non-aggregated cells to be seeded directly into the device, with equivalent numbers of cells seeded into each well (Fig. 2D). We found the seeding of cells through flow, to be inefficient in generating spheroids. For biomedical applications, the use of a pipette to seed cells in an exact location on a microfluidic chip is an advantage when accurately producing spheroids. This user-friendly approach is also compatible in conventional biology laboratories. For our experiments, the human colorectal adenocarcinoma cell line, HT29, was used to generate 3D spheroids. HT29 has been used extensively in 3D cell culturing based studies, and from previous multiple studies, we have found HT29 to form spheroidal cultures with relative ease^45–48^. As demonstrated in Figs. 2A and 8, we found the microfluidic platform presented here, enables HT29 cells to form uniformly sized spheroidal structures. 3D spheroids grew to a maximum of around 250 μm in diameter, dictated by the diameters of the spheroid-culturing wells. Typically, HT29 3D spheroids with ≥ 300 μm diameters, possess hypoxic and necrotic cores with physiological gradients of cell proliferation (outer rapidly proliferating and inner non-proliferating cells)^49^. It could therefore be postulated, that the HT29 3D spheroids grown in this study did not possess the in vivo like physiological gradients or hypoxic/necrotic cores.

Our device was additionally designed to streamline high-throughput chemosensitivity experiments and improve the overall efficiency of 3D cell culturing. To demonstrate this, we used the chemotherapeutic agent, 5-Fluorouracil, to treat the HT29 spheroids. As 5-FU is currently the primary anti-cancer drug used to treat colorectal cancers^50^, it makes for an excellent model system and demonstrates the relevant real-world application of our device. Following the culturing of 3D spheroids, treatment and analysis could be performed conveniently on-chip, through the perfusion of anti-cancer drugs and other culture media. Fluorescent imaging (qualitative analysis) using propidium iodide to visually highlight cell death in spheroids was complimented by the leakage and quantification of LDH in the supernatant (quantitative analysis), an indicator of cell death. Our study makes use of a single 3D cell culturing microfluidic platform, to administer an anti-cancer treatment and permit subsequent on-chip viability imaging confirmed by an LDH assay.

In our study, we demonstrated two different setups for performing chemosensitivity experiments in the microfluidic devices. The first utilised a single syringe pump to drive flow through one device at a time (Fig. 5). This limited-perfusion setup permits simple experiments to be conducted with a single experimental endpoint. However, experiments can also be performed with continuous flow as demonstrated by the second setup utilising a multichannel pump (Fig. 6). An advantage of continuous flow setup is that it better models the physiological flow of systemic circulation in the body. This second setup also enables multiple devices to be run simultaneously in parallel. Each channel is controlled independently, therefore flow through one microfluidic device can be switched off without disrupting flow through the rest of the channels or devices. In addition, the continuous flow setup was designed to be placed into a conventional tissue culturing incubator, permitting prolonged experiments (lasting multiple days) to be performed using existing laboratory infrastructure and environmental standards.

## Conclusion

In this study, we have developed and evaluated a 3D cell culturing microfluidic device. The device comprises a layer of PDMS with concave 3D cell culturing wells sandwiched between two layers of transparent PMMA, allowing for direct visualization. HT29 colorectal cancer cells in suspension were seeded into the wells through the central port on the top PMMA layer and subsequently formed into 3D spheroids. These spheroids were treated through one-time or continuous perfusion of the anti-cancer drug, 5-Fluorouracil, and the resulting cell viability was analysed using fluorescent microscopy and an LDH assay. The two perfusion regimes simulated different physiological flow conditions and enabled the evaluation of distinct methods of administering anti-cancer treatment. Finally, the microfluidic device described here offers a convenient, efficient and quick platform for generating, culturing, treating and analysing 3D cancer spheroids and will facilitate high-throughput screening of anti-cancer drugs on complex 3D in vitro models.

## Methods

### Designing and fabricating the microfluidic devices

A single microfluidic device consists of a central PDMS chip consisting of a single straight flow channel joining the inlet to outlet, with an array of 3D cell culturing wells located towards the centre. This is sandwiched between two layers of transparent PMMA allowing for direct optimal imaging. Schematic designs for the different parts of the flow device were drawn in SolidWorks (Dassault Systèmes, Vélizy-Villacoublay, France). To create the PDMS flow chips, the inverse design was printed as a reusable master mould on an EnvisionTEC 3D printer (EnvisionTEC GmbH, Gladbeck, Germany) in a photocurable acrylonitrile butadiene styrene (ABS) thermoplastic polymer. The well-forming hemispherical “pimples” in the master mould are 250 µm in diameter located in 5 × 5 array, on a pitch of 500 µm × 500 µm (Fig. 7). Chips were then cast from the master mould using PDMS with a release agent to aid removal. The PDMS flow chips were fixed between two layers of PMMA. The top layer of PMMA was fabricated with fluid inlet and outlet ports and a cell seeding port in the centre, while the bottom layer of PMMA serves as the device base (Fig. 1). The PMMA layers were machined from cast sheet, with inlet and outlet ports positioned to match the ends of the PDMS chip and machined to take standard UNF 1/4-28 flangeless fittings. An additional port was machined into the top layer of PMMA, located directly above the 3D cell culturing wells. The microfluidic device was fashioned together using four bolts, one in each corner, to hold the assembly together. Inlet and outlet ports were connected to tubing using flangeless fittings and a blanking plug was fitted to the central port to allow direct access to the cell culturing wells (Fig. 1). Following assembly, the microfluidic device was sterilised using a steam and high-pressured autoclave prior to the addition of cells.

### Preparation of cancer cells

The human colorectal adenocarcinoma cell line, HT29, was obtained from the European Collection of Authenticated Cell Cultures (Salisbury, UK). Cells were cultured in Roswell Park Memorial Institute (RPMI) 1640 Medium plus GlutaMAX (Gibco by Life Technologies, Paisley, UK) (without sodium pyruvate) supplemented with 10% (v/v) Fetal Bovine Serum (FBS) (Sigma-Aldrich) and maintained at 37 °C, 5% CO_2_ and 95% relative humidity (RH). Cell cultures were passaged weekly and seeded into 75 cm^2^ tissue culture flasks (Corning Inc., New York, USA). Complete cell media was changed once per week. 80–90% confluent cell cultures were used for the experiments.

### Culturing 3D tumour spheroids in a microfluidic device

Cells were washed and trypsinised to create a cell suspension of 2 × 10^7^ cells/mL in cell medium. 20 μL of cell suspension was deposited into the wells of assembled PDMS chips. The chips were then incubated and maintained under static conditions at 37 °C/5% CO_2_/95% RH. All cell media was changed daily using a pipette. Manual cell counting and acquisition of the images of spheroids was performed using the EVOS FL Imaging System (Life Technologies). For scanning electron microscopy (SEM), spheroids were cultured for 10 days then briefly washed with DPBS, fixed in 2.5% (v/v) glutaraldehyde for 20 min and subjected to increasing gradients of ethanol dehydration (25%, 40%, 60%, 80%, 90% and 100%). The top surface of the PDMS flow chip was then coated with 4 nm of iridium and imaged using a FEI Nova NanoSEM450 scanning electron microscope (Thermo Fisher).

### Anti-cancer treatment of 3D tumour spheroids in microfluidic devices

#### Culturing 3D HT29 spheroids

HT29 cells in suspension were deposited through the central port of the top PMMA layer into the wells of the PDMS flow chip in an assembled microfluidic device. Cell media was then flowed through the microfluidic device at 10 µL/min using an Aladdin Single-Syringe Pump (AL-2000, World Precision Instruments, Florida, USA). Following complete cell media perfusion through the device, the device was incubated (37 °C/5% CO_2_/95% RH) under static conditions.

#### 5-FU treatment (Limited-perfusion conditions)

Two different flow conditions were used to study chemosensitivity in 3D HT29 spheroids. For the first (limited-perfusion) condition, 48 h after seeding cells, 500 µM 5-FU was prepared in cell media and perfused through the microfluidic device at 20 µL/min using the single-syringe pump. This approach can only accommodate one device at a time. Following the perfusion of 500 µL of 5-FU (20 µL/min) completely through the microfluidic device, lasting 25 min, flow was stopped and the device was detached from the pump and placed back into incubation (37 °C/5% CO_2_/95% RH). The spheroids were incubated with 5-FU under static conditions for an additional 24 h.

#### 5-FU treatment (Continuous perfusion conditions)

For the second (continuous perfusion) condition, 48 h after seeding cells, 250 µM 5-FU was prepared in cell media and perfused continuously through five microfluidic devices in parallel at 20 µL/min for 5 days. An Ismatec IPC Low-Speed Digital Peristaltic Pump (Cole-Parmer Instrument Company Ltd, St. Neots, UK) with 8 independent channels, was used to drive continuous fluid flow. Microfluidic devices under continuous perfusion were kept in incubation (37 °C/5% CO_2_/95% RH) for the duration of the experiments.

### Evaluating cytotoxicity in 3D tumour spheroids

#### Lactate dehydrogenase (LDH) assay

Spent cell culture media supernatant was collected from the microfluidic device and stored at − 20 °C until the end of the continuous flow 5-FU treatment period. The LDH cytotoxicity assay was performed using the Pierce LDH Cytotoxicity Assay Kit (Thermo Fisher) and carried out as per the manufacturer’s instructions and guidelines. This method has been previously applied to evaluate the cell viability of tumour tissue specimens cultured on a microfluidic platform^51–53^.

#### Fluorescent imaging

5 μg/mL Hoechst 33342 and 1.3 μg/mL Propidium Iodide was prepared in cell media, and flowed into the device at 20 µL/min. Spheroids were incubated with the dyes for 30 min, washed with DPBS through flow and imaged using the EVOS FL Imaging System (Life Technologies). Imaging was carried out directly on-chip. We note that the spheroids were relatively well held in the individual wells. This is ideal when moving between different laboratory and measurement platforms.

### Image and statistical analysis

ImageJ (National Institute of Health, Maryland, USA) was used to analyse the transillumination and fluorescent images of 3D spheroids. The student t-test and one-way ANOVA were used to perform data statistical analyses using GraphPad Prism 8 (GraphPad Software, Inc., California, USA). *p* < 0.05 was considered to be statistically significant. Data is presented as the mean ± standard deviation.
